# Eosinophilic Variant of Granulomatosis With Polyangiitis

**DOI:** 10.7759/cureus.41633

**Published:** 2023-07-10

**Authors:** Rui L Fernandes, Mariana F Ornelas, Ana C Henriques, Maria I Correia, Teresa Faria

**Affiliations:** 1 Internal Medicine, Hospital Central do Funchal - Serviço de Saúde da Região Autónoma da Madeira, Entidade Pública Empresarial da Região Autónoma da Madeira (SESARAM, EPERAM), Funchal, PRT; 2 Endocrinology, Diabetes and Metabolism, Hospital Central do Funchal - Serviço de Saúde da Região Autónoma da Madeira, Entidade Pública Empresarial da Região Autónoma da Madeira (SESARAM, EPERAM), Funchal, PRT

**Keywords:** autoimmune-associated vasculitis, erythroderma, eosinophilic granulomatosis with polyangiitis, granulomatosis with polyangiitis, hypereosinophilia

## Abstract

Granulomatosis with polyangiitis (GPA) is a multisystemic necrotizing vasculitis with a special tropism to the respiratory tract and the kidneys. Although uncommon, GPA may be associated with hypereosinophilia and limited organ involvement. In these cases, American College of Rheumatology/European League Against Rheumatism (ACR/EULAR) classification criteria may be insufficient to establish the diagnosis. We described a limited form of GPA, hypereosinophilia, and predominant skin involvement.

## Introduction

Granulomatosis with polyangiitis (GPA), formerly known as Wegener granulomatosis, is a systemic vasculitis that preferentially involves medium and small-sized arteries, venules, and arterioles [1]​​​​. The histological features described in GPA consist of granulomatous inflammation around blood vessels and necrotizing vasculitis. Clinically, GPA is characterized by granulomatous inflammation of the upper and lower respiratory tract and pauci-immune glomerulonephritis. Although mild eosinophilia has been reported, significant eosinophilia is unusual with few cases reported in the literature [2]. In this circumstance, it can be challenging to distinguish GPA from eosinophilic granulomatosis with polyangiitis (EGPA) (formerly known as Churg-Strauss Syndrome) [3,4].​ We present a rare case of GPA with peripheral and tissue hypereosinophilia and a skin-predominant involvement. This case report was previously presented as a meeting abstract at the 20th European Congress of Internal Medicine, 9-11 June 2022.

## Case presentation

**Figure 1:**
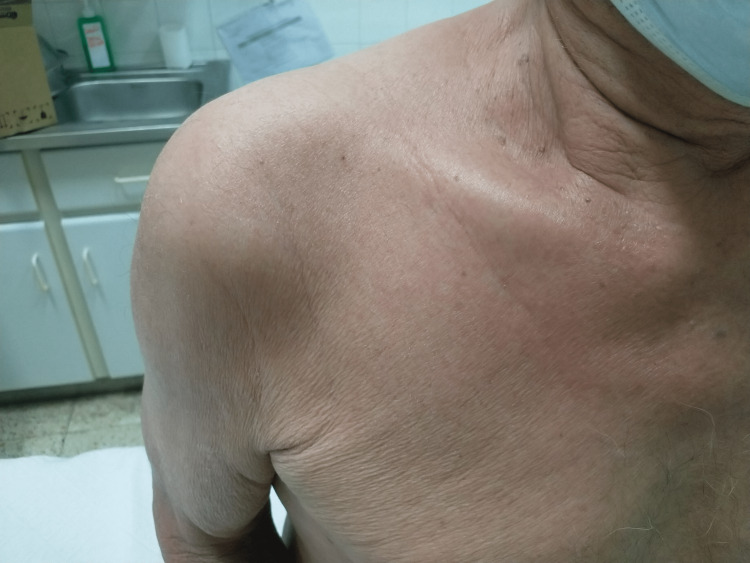
At physical examination, skin involvement was predominant. The skin was flushed, thick, and itchy.

A 71-year-old male was admitted for a progressive pruritic erythroderma, arthritis of large joints, hoarseness, fatigue, and weight loss (Figure 1). In addition, he had a 10-year history of hypereosinophilia of undetermined significance (HEus). There was no history of fever, sinusitis, asthma, or atopy.

His laboratory data at the emergency department showed a total white blood cell count of 21.6 x 10³/μL with 12.6 x 10³/μL eosinophils (58.2%). Reactive C-protein was 52.57 mg/L. Liver and renal function was normal, and urinary sediment did not show major alterations. Radiographs of chest, knees, and ankles were unremarkable. To clarify this hypereosinophilia, a broader blood study with auto-immunity and parasite infection screening was done. Immunoserology revealed positive c-anti-neutrophil cytoplasm antibodies (c-ANCA) and anti-proteinase 3 (anti-PR3) antibody was 31.4 U/L. Anti-nuclear antibodies and rheumatoid factor were negative. Immunoglobulin E was elevated (> 5000 kU/L) (Table 1). Stool microscopy identified rare Strongyloides stercoralis eggs. Blood cultures were negative.

**Table 1:** Results of auto-immunity at admission ANCA: Anti-neutrophil cytoplasmic antibody

Auto-immunity	Admission
Ac. ANCA, result	Positive
Ac. ANCA, title	1/40
Ac. ANCA, pattern	c-ANCA
c-ANCA	31.4 U/L
p-ANCA	0.3 U/L
Ac. Anti-CCP	0.3
IgE total	>5000 kU/L

**Figure 2:**
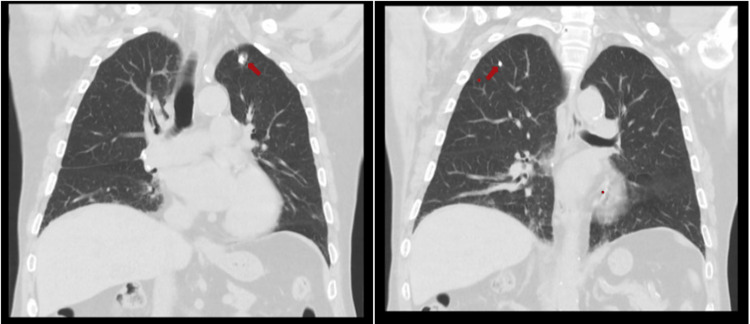
Chest CT. Red arrows show some of the granulomas. CT scan reassessment after beginning steroids did not show modifications.

Computed tomography (CT) scans of chest and abdomen revealed multiple granulomas in left and right lung apexes (Figure 2). Laryngoscopy was performed and showed left vocal cord palsy, in probable relation with recurrent laryngeal nerve compression by lung granuloma. Skin biopsy was compatible with angiocentric dermatitis with surrounding tissue hypereosinophilia. Later, the cranial magnetic resonance did not show any nasal or paranasal sinus mucosa alteration. Cardiac ultrasound was unremarkable.

Due to the suspicion of ANCA-associated vasculitis (AAV), the patient was started on prednisolone 1 mg/kg/day after deworming with one single dose of ivermectin. Deworming was complete and was not associated with any clinical or laboratory improvement. After starting prednisolone, there was a quick normalization of eosinophilia, as well as an improvement in constitutional symptoms and reduction of redness and pruritus of the skin. After two years of follow-up, the patient is 2.5mg prednisolone-dependent.

## Discussion

In the present case report, the patient had peripheral hypereosinophilia for several years without clinical repercussions, also known as HEus. More recently, he developed a pruritic erythema and concurrently, aggravated hypereosinophilia and hyper-IgE. The immunoserology profile and histology results are highly suggestive of AAV with tissue hypereosinophilia. In this clinical scenario, EGPA and the eosinophilic variant of GPA emerge as the main diagnoses.

These diagnoses are two distinct entities within the spectrum of eosinophilic vasculitis. While they share certain clinical and pathological features, they also exhibit significant differences. EGPA is characterized by asthma, eosinophilia, and systemic vasculitis. It typically affects multiple organ systems, including the lungs, skin, heart, gastrointestinal tract, and peripheral nerves. EGPA is often associated with allergic rhinitis, sinusitis, and nasal polyposis. Diagnostic criteria for EGPA include asthma, eosinophilia, systemic vasculitis, and histopathological evidence of eosinophilic inflammation. EGPA is commonly identified by the presence of anti-myeloperoxidase (anti-MPO) ANCA [5].

On the other hand, GPA is a necrotizing granulomatous vasculitis that primarily affects small- to medium-sized vessels. It predominantly involves the upper and lower respiratory tract and kidneys, resulting in sinusitis, pulmonary nodules, and glomerulonephritis. GPA is highly associated with the presence of anti-proteinase 3 (PR3) ANCA [6].

The eosinophilic variant of GPA represents a subtype of GPA characterized by prominent eosinophilic infiltration in affected organs. This subtype was described for the first time in 1988 [7]. Since then, a few case reports have been describing atypical presentations of GPA with preferential organ involvement [8-10]. According to the French Vasculitis Study Group, hypereosinophilia can be detected in about one-quarter of GPA patients at diagnosis [11]. This subset of patients appears to have a higher disease activity at diagnosis and more skin and neurological involvement.

**Figure 3:**
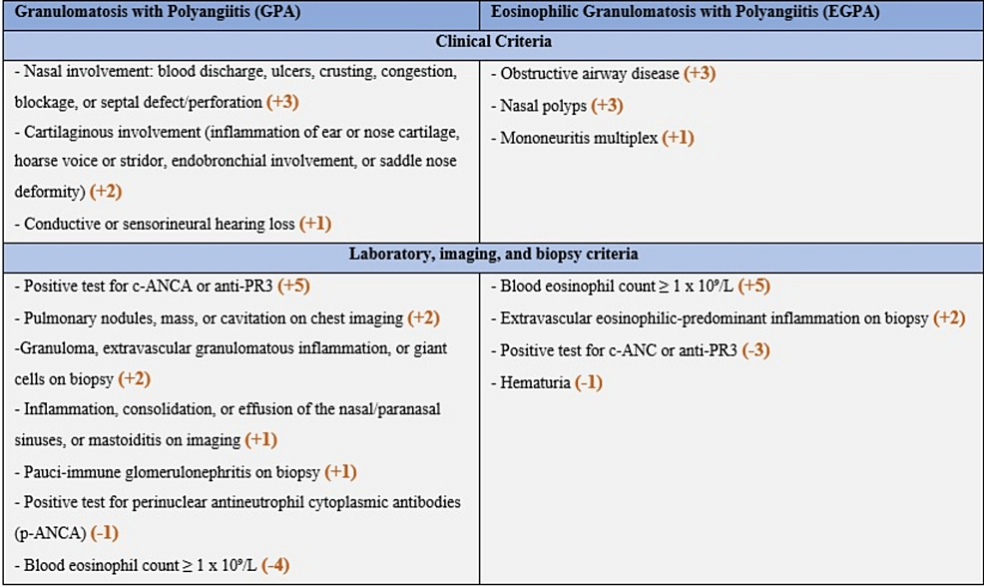
The 2022 American College of Rheumatology (ACR)/European League Against Rheumatism (EULAR) classification criteria to GPA and EGPA. A score ≥ 6 is needed for classification of EGPA and ≥ 5 for GPA. ANCA: Anti-neutrophil cytoplasmic antibody

In this case report, we suspect that we are dealing with an eosinophilic variant of GPA with a predominant involvement of the skin. Our hypothesis is supported by the absence of obstructive airway disease typically observed in EGPA and the presence of anti-proteinase 3 (PR3) ANCA, which is highly specific to GPA but rarely seen in EGPA (Figure 3). 

Other hypotheses, like asthma and allergic bronchopulmonary aspergillosis, are implausible. The patient has no personal or family history of atopy. On the other hand, it has no clinical manifestations or CT scan changes compatible with ABPA. Lastly, although infrequent, elevated levels of IgE have been described in GPA and parasite eradication did not change IgE levels [12].

Furthermore, different classification criteria and scoring systems are employed to aid in diagnosis, assess disease severity, and guide treatment decisions. One of the most established is the “Revised 2011 Five Factor Score (FFS)”, which is a primarily prognostic tool based on factors such as age > 65 years, cardiac symptoms, gastrointestinal involvement, renal insufficiency, and absence of ear-nose-throat (ENT) manifestations [13]. Our patient has a score of 2 points (age > 65 years and absence of ENT manifestations) which is considered to have a poor prognosis and necessitates the need for a more aggressive immunosuppression [14].

In this particular case, the decision to initiate remission induction therapy solely with corticosteroids was primarily based on the patient's clinical stability. Due to the favorable response observed, it was deemed appropriate to continue with this treatment strategy. Presently, the patient is receiving a dose of 2.5mg of prednisolone. Previous attempts to discontinue the medication resulted in a subsequent rise in eosinophil levels, prompting the decision not to suspend it again. In summary, EGPA and the eosinophilic variant of GPA represent distinct subsets within the spectrum of eosinophilic vasculitis. A comprehensive evaluation, including clinical, serological, and histopathological findings, is crucial for accurate diagnosis and appropriate management of these conditions.

## Conclusions

In conclusion, GPA is a vasculitis with a special tropism to the lungs and kidneys and rarely associated with hypereosinophilia. However, the clinician should be aware of a subset of GPA with hypereosinophilia, sharing aspects with EGPA, and limited organ involvement. In our clinical case, the patient has a predominant cutaneous vasculitis with peripheral and tissue hypereosinophilia, and the diagnosis of GPA was supported by histology and immunoserology patterns.
